# Knowledge and Awareness of Cervical Cancer among HIV-Infected Women in Ethiopia

**DOI:** 10.1155/2016/1274734

**Published:** 2016-10-30

**Authors:** Netsanet Shiferaw, Mohamad I. Brooks, Graciela Salvador-Davila, Shumet Lonsako, Konjit Kassahun, Jodi Ansel, Chidude Osakwe, Teklu Weldegebreal, Ismael Ahmed, Mengistu Asnake, Paul D. Blumenthal

**Affiliations:** ^1^Pathfinder International, Addis Ababa, Ethiopia; ^2^Pathfinder International, Watertown, MA, USA; ^3^Boston University School of Public Health, Boston, MA, USA; ^4^United Methodist Committee on Relief (UMCOR), New York City, NY, USA; ^5^University of Maryland, School of Medicine, Baltimore, USA; ^6^Massachusetts General Hospital, Center for Global Health, Boston, MA, USA; ^7^The United States Center for Disease Control and Prevention (CDC), Addis Ababa, Ethiopia; ^8^Stanford Program for International Reproductive Education and Services (SPIRES), Stanford University School of Medicine, Stanford, CA, USA

## Abstract

*Introduction*. Cervical cancer is one of the leading causes of cancer death among Ethiopian women. Low awareness of cervical cancer, in combination with low health care seeking behavior, is a key challenge for cervical cancer prevention. This study assessed the knowledge of cervical cancer among HIV-infected women in Ethiopia.* Methods*. A facility-based cross-sectional survey was conducted from August to September 2012 among HIV-infected women between 21 and 49 years of age. Basic descriptive statistics were performed using SPSS.* Results*. A total of 432 HIV-infected women participated in this study. About 71% of participants had ever heard of cervical cancer. Among women who had ever heard of cervical cancer, 49% did not know the cause while 74% were able to identify at least one risk factor for cervical cancer. Only 33% of women were able to correctly address when women should seek care and 33% identified at least one treatment option for cervical cancer.* Conclusion*. This study revealed that knowledge about cervical cancer was generally low, in particular for health care seeking behavior and treatment of cervical cancer. Health awareness programs should be strengthened at both community and health facility levels with emphasis highlighting the causes, risk factors, care seeking behaviors, and treatment options for cervical cancer.

## 1. Introduction

In 2008, one in every six cancers worldwide was caused by an infection that could be prevented or treated [1]. Human papillomavirus (HPV) is responsible for 99% of cervical cancer and accounts for approximately half of the infection-related burden of cancer in women [1, 2]. In 2012, about 85% of the global burden of cervical cancer was reported from developing countries with Sub-Saharan Africa as the region with the highest incidence of cervical cancer in the world [2–4].

In Ethiopia, an estimated 19,836 new cases (26.4 per 100,000 women) and 16,283 deaths (18.4 per 100,000 women) of cervical cancer were reported in 2012 [3]. These figures most likely underestimate the actual number of cases given the low level of awareness for cervical cancer, limited access to cervical cancer screening and treatment services, and lack of a representative population-based cancer registry. Historically, Ethiopia has invested little effort in cancer awareness as maternal health and other communicable diseases have been targeted as key health priorities by the Federal Ministry of Health (FMOH) [5, 6].

Lack of effective screening programs aimed at detecting and treating precancerous cervical conditions, low public awareness, and overall low health care seeking behavior of women are key challenges for cervical cancer prevention (CCP) in developing countries [4, 7–9].

Access to cervical cancer screening was extremely limited for the majority of women in Ethiopia until the Single Visit Approach (SVA) for CCP service was introduced by Pathfinder International in 2009. The CCP project, named* Addis Tesfa* (which translates to* New Hope*), introduced the SVA to women infected with human immunodeficiency virus (HIV) in a phased approach in 14 public PEPFAR-affiliated health facilities. The SVA employed Visual Inspection of the cervix with Acetic acid wash (VIA) and offered immediate treatment of precancerous cervical lesions with cryotherapy [5, 10–13]. Women infected with HIV are at higher risk for precancerous lesions and are more likely to progress to invasive cervical cancer compared with uninfected women [14–16]. As a result, the FMOH of Ethiopia supported the integration of CCP services within selected HIV/AIDS centers. This study was designed to assess the level of knowledge and awareness of cervical cancer among HIV-infected women in Ethiopia who visited the* Addis Tesfa* supported health facilities.

## 2. Materials and Methods

### 2.1. Study Design, Sites, and Period

This descriptive cross-sectional study took place in five public health facilities providing SVA services from August to September 2012. The selected sites were all centers of excellence and were either teaching or referral hospitals within the* Addis Tesfa* project. These sites were located in urban settings in each of the five major regions in Ethiopia: Tigray, Amhara, Oromia, Southern Nations, Nationalities, and People's Region (SNNPR), and Addis Ababa City Administration.

### 2.2. Study Population

Women infected with HIV between the ages of 21 and 49 years, visiting the selected facilities during the study period, and consenting to be interviewed were included in the study. Women who had a history of cervical cancer and/or hysterectomy were excluded.

### 2.3. Sample Size Determination and Sampling Method

A sample size of 442 was calculated using a single population proportion formula (*z* ^2^ *∗P*(1 − *P*)/*d* ^2^) with 15% nonresponse rate, population proportion of 50%, significance level of 0.05, and a confidence level of 95%. All five public health facilities that provided the SVA service were included as study sites. Enrollment to the study was based on quota sampling which was proportional to each facility's HIV client load. All eligible clients who presented for follow-up visit at the HIV/AIDS center in each of the five health facilities were approached for study enrollment. Clients who gave consent for participation were enrolled consecutively until the target sample size was met for each study site.

### 2.4. Data Collection Instrument

A structured questionnaire was developed from similar cervical cancer knowledge and awareness studies implemented in developing countries [17–20]. Prior to study initiation, the questionnaire was translated into three local languages (Amharic, Oromiffa, and Tigrigna) and pilot tested on 44 women in one study site. The questionnaire captured information on sociodemographic characteristics and asked questions associated with the knowledge and awareness of cervical cancer.

### 2.5. Ethical Considerations

Data collectors and supervisors received two days of training which included study overview, informed consent process, and detailed review and administration of the study questionnaire. Written consent was obtained from all study participants and ethical clearance was granted from the institutional review boards of the Ministry of Science and Technology of Ethiopia and the United States Centers for Disease Control and Prevention (CDC).

### 2.6. Data Collection and Analysis

Data quality was checked at all levels of data collection and aggregation. The level of cervical cancer knowledge was measured by adding up the total score from ten questions on cause, risk factors, and prevention and treatment options for cervical cancer among HIV-infected women who had ever heard of cervical cancer. Response to each question was categorized into correct (1) and incorrect (0) and the combined score was calculated. The median of the ten questions was taken as a cutoff point to assess the knowledge level; participants who scored above the median were considered to be knowledgeable about cervical cancer while those who scored at or below the median were considered to be less knowledgeable. Basic descriptive statistics were performed using SPSS (version 20).

## 3. Result

### 3.1. Sociodemographic Characteristics

A total of 432 women infected with HIV participated in this study (Table 1). The study had a participant response rate of 98%. The median age was 33 years with an age range from 21 to 49 years (M = 34.1, SD = 4.8). About four out of ten (39.6%) women were married and 33.1% had no formal education. One-third (32.6%) of participants were housewives and the majority (85%) resided in urban areas. About half of the participants were from health facilities in the Tigray (24.3%) and Amhara (23.1%) Regions. Six out of ten women who participated in this study came to the health facility as part of routine HIV/AIDS follow-up care (Table 1).

### 3.2. Cervical Cancer Cause, Risk, and Source of Information

About seven out of ten (71.3%) participants had ever heard of cervical cancer and 57.3% stated that the main source of information for cervical cancer was from the health facility (Table 2). Among women that had ever heard of cervical cancer, about half (49.0%) did not know the cause of cervical cancer. About three out of ten (29.2%) participants did not know that cervical cancer was a sexually transmitted illness (STI) and 26.3% were unable to name a single risk factor for cervical cancer. Slightly over half (54.9%) of participants were able to mention HIV as a risk factor for cervical cancer. Among this group of HIV-infected women, about half (50.3%) considered themselves to be at risk for cervical cancer (Table 2).

### 3.3. Cervical Cancer Prevention (CCP) and Treatment

Most women believed that cervical cancer is a preventable (75.3%) and treatable (66.2%) health problem (Table 3). A majority of women (85.4%) acknowledged that cervical cancer can be prevented through routine screening for and treatment of precancerous lesions. About four out of ten (43.2%) respondents said that women should seek care for cervical cancer only if she showed signs and symptoms in her reproductive organs; about a quarter (25.6%) had no idea about when women should seek care related to cervical cancer. Two-thirds of women (66.6%) were unable to mention any treatment options for cervical cancer and a higher proportion of women (89%) did not know the treatment options for precancerous lesions. The lack of information on cervical cancer and available preventive services (58.4%) and fear of test results (43.8%) were some of the most common opinions on why women do not get screened for cervical cancer (Table 3).

### 3.4. Comprehensive Knowledge of Cervical Cancer

The median score used to determine the overall cervical cancer knowledge was two (M = 2.39, SD = 1.73) with participant scores ranging from zero to eight (out of ten possible points). Based on this median cutoff score, less than half of the participants (43.8%) were considered to be knowledgeable about cervical cancer. Fifty-two women (16.9%) who had ever heard of cervical cancer did not respond correctly to any of the ten knowledge questions (Table 4).

## 4. Discussion

This is the first study to our knowledge that investigated cervical cancer knowledge and awareness among HIV-infected women in Ethiopia. The characteristics of study participants differed from the general population of women in Ethiopia. Compared to the results from the 2011 Ethiopian Demographic and Health Survey (DHS), women enrolled in this study were more educated (no formal education: 33.1% [study] versus 50.8% [DHS2011]), were primarily comprised of Orthodox Christians (Orthodox religion: 79.6% [study] versus 47.5% [DHS2011]), were more likely to be divorced, separated, or widowed (divorced/separated/widowed: 45.6% [study] versus 10.6% [DHS2011]), and were living in urban settings (urban: 85% [study] versus 32.5% [DHS2011]) [21]. These differences are expected as the study was not designed to capture nationally representative data.

Increasing knowledge and awareness of cervical cancer plays a major role in improving health care seeking behavior for CCP services [19]. The results of this study are consistent with published literature of cervical cancer knowledge and awareness among women in Sub-Saharan African countries [17–19, 22–25]. The findings show that 71% of HIV-infected women in this study had ever heard of cervical cancer, which is comparable to the results found among women in the community of Gondar (79%) in the region of Amhara in northeast Ethiopia [18]. Between the two studies conducted in Ethiopia, women's awareness of cervical cancer—as defined by having heard of cervical cancer—was found to be slightly higher in the community-based study by Getahun et al. compared to this facility-based study conducted among HIV-infected women. As the Getahun et al. study was implemented in one specific region, it appears that women in Gondar may have had higher awareness of key health topics, including cervical cancer, compared to other areas of the country. This theory may be supported by the findings from this study which showed that a large proportion of women from the health facility in Amhara Region had better knowledge of cervical cancer compared to women from other health facilities. The difference in knowledge among health facilities in this particular study may be related to the intensity of health education programs across the different regions of the* Addis Tesfa* project. Project documents have shown that implementation of demand creation activities occurred more frequently in Amhara and SNNPR compared to other regions of the project.

The findings from this study support past research that has shown that awareness of cervical cancer does not translate into comprehensive knowledge of cervical cancer [18, 22, 23]. The results indicate that although 71% of women in this study had ever heard of cervical cancer, only 44% had cervical cancer knowledge above the median score. Similar studies have noted that adequate knowledge of cervical cancer remains relatively low, ranging from 37% in Ghana [22], to 31% in Ethiopia [18], to as low as 23% in Nigeria [23]. Knowledge of cervical cancer may be higher in this study because of the recruitment of HIV-infected women who were more likely to have prior exposure and interaction with the health system. The participants recruited in this study were all HIV-infected women coming to an HIV/AIDS center for either a routine HIV follow-up visit (60%), treatment for a current illness (7%), or getting a cervical cancer screening (33%).

Among women in this study that had ever heard of cervical cancer, over half (57%) of participants stated that the source of information for cervical cancer was from the health facility while close to a quarter (23%) mentioned the media. In the community-based survey conducted by Getahun et al. [18], the predominant source of information was reversed—61% of women stated that their source of cervical cancer information was the media while only 35% mentioned health facilities or health providers. The difference in the source of information may be a result of the study setting. Women recruited from the community may be more likely to receive cervical cancer information from the media while women recruited from health facilities may be more likely to receive this information from health professionals.

Having information on the cause and risk factors of cervical cancer is helpful for a woman to take preventive action. In this regard, only 40% of women in this study mentioned that a virus is the primary cause of cervical cancer. This result was higher than the study among HIV-infected women in Lagos, Nigeria, where only 8% knew that HPV causes cervical cancer [8]. In both countries, understanding disease transmission was extremely low; 15% of this study's participants linked the HPV transmission to sexual contact while 18% knew that HPV is contracted via sexual intercourse in Nigeria [8]. A higher proportion of women in this study (55%) knew that HIV was a risk factor for cervical cancer compared to the study conducted in Zimbabwe among HIV-infected women (27%) [20]. In a similar study conducted among educated women at the University of Ghana, 77% of women were able to identify at least one risk factor for cervical cancer [22]. The findings from these studies suggest that women with higher educational attainment, or those recruited from health facilities, were more likely to identify risk factors associated with cervical cancer.

Although close to three-quarters (74%) of women in this study were able to identify at least one risk factor for cervical cancer, only one-third (33%) were able to identify at least one treatment option for cervical cancer. In the study conducted in northeast Ethiopia, 66% of women in the community were able to identify at least one treatment option [18]. Even though the exact same questions were used in both surveys, women in the community in Gondar seem to know more about cervical cancer treatment options compared to HIV-infected women visiting health facilities throughout this study setting at different regions of Ethiopia. This difference may be due to the exposure of prior health and cancer awareness programs in the community of Gondar which may increase overall cervical cancer knowledge within this population.

In resource-constrained settings, it is important to note the ethical considerations of raising disease awareness in communities where screening and treatment are not readily available. Awareness raising programs in the absence of services can be controversial and can erode public confidence in the health system, with the potential to negatively impact other preventative public health programs in a country. Health programs designed to increase awareness should be combined with initiatives that also provide the appropriate screening, diagnostic, and treatment services.

Several limitations exist in this study. As this study was conducted at an HIV/AIDS center, there is potential for selection bias as women who come to these health facilities are likely to have better health seeking behavior and health information compared to women from the general community. In addition, this study used quota sampling, a nonprobability sampling technique which does not allow calculation of sampling error; therefore statistical inferences from this sample were not possible. Social desirability bias might also be a concern due to the sensitive nature of this topic. Sexual and reproductive health topics are sensitive issues in a conservative community; therefore, participants may answer a survey question in a way that will be viewed more favorably by the research team.

## 5. Conclusion

The results of this study revealed that even though most HIV-infected women have heard of cervical cancer, the knowledge and awareness of cervical cancer among women attending clinical care services was generally low. In particular, the knowledge gaps were observed on care seeking behavior and treatment of cervical cancer in this study population. These knowledge gaps affect women's care seeking behavior and are detrimental to the national effort towards cervical cancer prevention, treatment, and control. Cervical cancer awareness should be strengthened both at the community and health facility level. Special emphasis must be placed on the causes, risk factors, care seeking behavior, and treatment options for cervical cancer. A successful cervical cancer prevention, treatment, and control program not only will focus on awareness raising efforts, but should also be combined with programs to ensure access to cervical cancer screening, diagnostic, and treatment services.

## Figures and Tables

**Table 1:** Sociodemographic characteristics of HIV-infected women enrolled in this study, Ethiopia, August to September 2012.

Variable	Number (*n* = 432)	%
Age		
Below 30	26	6.0
30–34	207	47.9
35–39	121	28.0
40 and above	78	18.1
Educational status		
No formal education	143	33.1
Elementary	162	37.5
Secondary	96	22.2
Tertiary education	31	7.2
Marital status		
Single	64	14.8
Married	171	39.6
Divorced/separated	104	24.1
Widowed	93	21.5
Occupation		
Housewife	141	32.6
Business work	118	27.3
Government employee	51	11.8
Self-employed (daily laborer, farmer)	74	17.1
Work at private organization or in small business group	23	5.3
Other (house servant, lives with family)	25	5.8
Religion		
Orthodox	344	79.6
Protestant	45	10.4
Muslim	34	7.9
Other religions	9	2.1
Geographic location		
Health facility 1 (Addis Ababa City Administration)	80	18.5
Health facility 2 (Tigray)	105	24.3
Health facility 3 (Amhara)	100	23.1
Health facility 4 (Oromia)	70	16.2
Health facility 5 (Southern Nations, Nationalities, and People's Region [SNNPR])	77	17.8
Residence		
Urban	367	85.0
Semiurban	28	6.5
Rural	37	8.5
Reason that brought them to the facility		
Routine HIV/AIDS follow-up care	259	60.0
To get cervical cancer screening test	141	32.6
To get treatment for current illness	32	7.4

**Table 2:** Awareness and knowledge of cervical cancer among HIV-infected women involved in the study, Ethiopia, August to September 2012.

Variable	Number	%
Ever heard about cervical cancer		
Yes	308	71.3
No	124	28.7
*Total*	*432*	*100*
Source of information for cervical cancer (*n* = 308)		
Health facility	176	57.3
Media	70	22.8
Friends	29	9.4
Relatives	33	10.7
The primary cause of cervical cancer (*n* = 308)		
I don't know/cause unknown	151	49.0
Infection by a germ/virus	122	39.6
God's punishment	16	5.2
Other (related and non-related risk factors)	19	6.2
Women potentially at risk of developing cervical cancer (*n* = 308)		
Women who have ever had sex are at risk	47	15.3
All women are at risk	108	35.1
Women with poor hygiene	84	27.3
I don't know	69	22.4
Is cervical cancer a sexually transmitted illness? (*n* = 308)		
Yes	194	63
No	24	7.8
I don't know	90	29.2
Who is most at risk for cervical cancer? (*n* = 308)^*∗*^		
Women infected with HIV	169	54.9
Women with multiple sexual partners	102	33.1
Women exposed to repeated STIs	61	19.8
Women who have sex before 18 years of age	42	13.6
Women who bear too many children	45	14.6
A wife of a man who had multiple sexual partners	45	14.6
Women who had a mother or sister with cervical cancer	11	3.6
Don't know	81	26.3
Do you consider yourself at risk for cervical cancer? (*n* = 308)		
Yes	155	50.3
No	133	43.2
I don't know	20	6.5

^*∗*^Percent exceeds 100% as multiple answers are possible.

**Table 3:** Awareness and knowledge of cervical cancer prevention (CCP) and treatment among HIV-infected women involved in the study, Ethiopia, August to September 2012.

Variable	Number (*N* = 308)	%
Is cervical cancer an avoidable/preventive health problem?		
Yes	232	75.3
No	29	9.4
I don't know	47	15.3
Is cervical cancer a treatable health problem?		
Yes	204	66.2
No	34	11.0
I don't know	70	22.7
Can cervical cancer be prevented through routine screening and precancerous lesion treatment?		
Yes, agree	263	85.4
No, disagree	45	14.6
When should a woman seek care related to cervical cancer?		
Once she is sexually active, she should be scheduled for screening	96	31.2
She needs to visit a health care facility only if she has a sign or symptom in her reproductive organs	133	43.2
I have no idea	79	25.6
What treatment do you know for women diagnosed with cervical cancer?^*∗*^		
Surgery	28	9.1
Chemotherapy/radiation	61	19.8
No treatment, just waiting for death	19	6.2
I don't know	205	66.6
What treatment do you know for women diagnosed with precancerous lesion?		
Cryotherapy (treatment applied on the cervix that kills the cancer cell)	9	2.9
No treatment, just waiting for death	13	4.2
Others (counseling, vaccination, etc.)	12	3.9
I don't know	274	89.0
What do you think is the reason that some women don't want to get screened?^*∗*^		
Fear of test result	135	43.8
Lack of information on cervical cancer and available preventive service	180	58.4
People are shy to talk about this type of issue	97	31.5
Many feel that they are healthy (low risk perception)	86	27.9
Because of rumors and myths about the test and treatment	49	15.9
Afraid of exposing their reproductive organ for examination and unwilling to receive care from male providers	40	13.0
Women don't have enough money to travel to the service	38	12.3

^**∗**^Multiple answers are possible.

**Table 4:** Overall knowledge of cervical cancer among HIV-infected women involved in the study, Ethiopia, August to September 2012.

Correct answers used to measure cervical cancer knowledge	Number (*N* = 308)	%
(1) The primary cause of cervical cancer is a germ/virus	122	39.6
(2) Women who have sex before 18 years of age are at high risk for cervical cancer	42	13.6
(3) Women exposed to repeated sexually transmitted illness are at high risk for cervical cancer	61	19.8
(4) Women infected with HIV are at high risk for cervical cancer	169	54.9
(5) Women with multiple sexual partners are at high risk for cervical cancer	102	33.1
(6) A wife of a man who had multiple sexual partners is at high risk for cervical cancer	45	14.6
(7) Women who have a mother or sister with cervical cancer are at high risk for cervical cancer	11	3.6
(8) Women should seek care related to cervical cancer once they become sexually active	96	31.2
(9) Cryotherapy is a treatment option for women diagnosed with precancerous cervical lesion	9	2.9
(10) Treatment options for women diagnosed with cervical cancer include chemotherapy, radiation, and surgery	79	25.6

Overall cervical cancer knowledge level	Number (*N* = 308)	%

Women knowledgeable about cervical cancer (cumulative knowledge score > 2)	135	43.8
Women less knowledgeable about cervical cancer (cumulative knowledge score ≤ 2)	173	56.2

## Acronyms

AIDS: Acquired immunodeficiency syndrome
CCP: Cervical cancer prevention
CDC: Centers for Disease Control and Prevention
FMOH: Federal Ministry of Health
HIV: Human immunodeficiency virus
HPV: Human papilloma virus
SNNPR: Southern Nations, Nationalities, and People's Region
STI: Sexually transmitted illness
SVA: Single Visit Approach.
